# Disease Phenotypes in a Mouse Model of RNA Toxicity Are Independent of Protein Kinase Cα and Protein Kinase Cβ

**DOI:** 10.1371/journal.pone.0163325

**Published:** 2016-09-22

**Authors:** Yun K. Kim, Ramesh S. Yadava, Mahua Mandal, Karunasai Mahadevan, Qing Yu, Michael Leitges, Mani S. Mahadevan

**Affiliations:** 1 Department of Pathology, University of Virginia, Charlottesville, Virginia, United States of America; 2 The Biotechnology Centre of Oslo, University of Oslo, Oslo, Norway; Colorado State University, UNITED STATES

## Abstract

Myotonic dystrophy type 1(DM1) is the prototype for diseases caused by RNA toxicity. RNAs from the mutant allele contain an expanded (CUG)_n_ tract within the 3' untranslated region of the dystrophia myotonica protein kinase (DMPK) gene. The toxic RNAs affect the function of RNA binding proteins leading to sequestration of muscleblind-like (MBNL) proteins and increased levels of CELF1 (CUGBP, Elav-like family member 1). The mechanism for increased CELF1 is not very clear. One favored proposition is hyper-phosphorylation of CELF1 by Protein Kinase C alpha (PKCα) leading to increased CELF1 stability. However, most of the evidence supporting a role for PKC-α relies on pharmacological inhibition of PKC. To further investigate the role of PKCs in the pathogenesis of RNA toxicity, we generated transgenic mice with RNA toxicity that lacked both the PKCα and PKCβ isoforms. We find that these mice show similar disease progression as mice wildtype for the PKC isoforms. Additionally, the expression of CELF1 is also not affected by deficiency of PKCα and PKCβ in these RNA toxicity mice. These data suggest that disease phenotypes of these RNA toxicity mice are independent of PKCα and PKCβ.

## Introduction

Myotonic dystrophy type 1 (DM1) is a slowly progressing and highly variable multisystemic disorder. It is characterized by wasting of muscles and weakness. DM1 is caused by an expanded (CTG)n repeat in the 3’-untranslated region (UTR) of the DM protein kinase (DMPK) gene [1–3]. The mutant RNA forms RNA foci, which alter the activity of RNA binding proteins such as CELF1 and muscleblind-like 1(MBNL1)[4, 5]. MBNL proteins can co-localize with the RNA foci [6–8], and the prevailing model of DM1 pathogenesis invokes sequestration of these proteins by the mutant *DMPK* mRNA [4]. Strong evidence for the role of MBNL proteins in DM1 pathogenesis has been obtained through mouse knockout models of the various *Mbnl* genes [9–13]. In contrast, CELF1 levels are reportedly increased in myoblasts [14], in the heart [15], and skeletal muscles from DM1 patients [16]. Thus, mouse models have utilized over-expression of CELF1 and demonstrated DM1 related phenotypes such as muscle histopathology and cardiac defects [17–19]. Proposed molecular mechanisms of increased CELF1 invoke signaling pathways mediated by PKCs and/or glycogen synthase kinase 3 beta (GSK3β) [20–22]. Consistent with this idea, inhibitors of PKC and GSK3β were able to rescue some of the salient phenotypes in mouse models of RNA toxicity [21, 23].

The protein kinase C (PKC) family comprising many isoforms, phosphorylates serine and threonine residues in many target proteins [24]. Different PKC isoforms are expressed in skeletal muscle, including the classical isoform, PKCα [25]. PKCα is the predominant isoform in skeletal muscle, whereas PKCβ and PKCγ are expressed at very low levels [26]. The role of PKC in RNA toxicity in skeletal muscle is not clear, but it has been investigated in a cardiac specific mouse model using pharmacological inhibitors that were effective in improving cardiac phenotypes [23]. Previously, we have shown increased CELF1 expression in our inducible/reversible DM5 mouse model of RNA toxicity and that CELF1 levels are responsive to the presence of the toxic RNA [27]. In addition, we demonstrated that the levels of CELF1 in skeletal muscle correlated with skeletal muscle histopathology in the mouse model and in tissues from patients with DM1 [28]. Of note, genetic deletion of *Celf1* in the DM5 mice resulted in mild improvement of muscle histology [28]. Since increased CELF1 levels are thought to be due to activated PKC, we investigated the role of PKC in the skeletal muscle phenotypes of our RNA toxicity mice using a genetic approach.

## Results

### Phenotypic effects of *Prkca*^*-/-*^*/Prkcb*^*-/-*^ double knockout in the RNA toxicity mice

Using our inducible/reversible DM5 mouse model of RNA toxicity, we have shown that induction of toxic RNA expression (with 0.2% doxycycline in drinking water) results in many features of DM1 includes myotonia, cardiac conduction abnormalities, abnormal muscle pathology, and RNA splicing defects [27]. In this model, CELF1 is increased in the skeletal muscle, but not in the heart [27]. We also showed that deletion of *Celf1* in this model results in mild improvement in skeletal muscle histopathology [28]. To assess the role of PKCα in regulating CELF1 levels and the phenotypes in these RNA toxicity mice, preliminary experiments were done using *Prkcα* knockout mice (*Prkca*^*tm1Jmk*^) obtained from Dr. J. Molkentin [29]. The DM5/ *Prkca*^*tm1Jmk +/+*^, DM5/ *Prkca*^*tm1Jmk +/-*^, *and* DM5*/ Prkca*^*tm1Jmk -/-*^ mice were normal before induction of RNA toxicity. After induction with 0.2% doxycycline (w/v), all the mice developed severe myotonia and similar degrees of advanced cardiac conduction abnormalities at two weeks post-induction. We found no significant differences between the groups in terms of survival, running distance, and grip strength after one and two weeks of induction (S1 Fig).

We also obtained another *Prkca* (*Pkcα)* knockout mouse as well as a *Prkcb* (*Pkcβ*) knockout mouse from Dr. M. Leitiges [30, 31]. The rest of the experiments were done with these lines bred with the DM5 mice to generate double knockout mice in the RNA toxicity background. Due to the high severity of the phenotypes in the DM5 mice, including severe cardiac conduction abnormalities that led to mortality in the preliminary experiments, we tried various lower concentrations of doxycycline. We found that 0.02% doxycycline led to robust induction of myotonia without severe cardiac conduction abnormalities or increased mortality. This resulted in 2–3 fold induction of toxic RNA expression in the skeletal muscle and no induction in the heart (S2 Fig). This correlated with the absence of severe cardiac conduction abnormalities at 2, 4, 6, and 8 weeks after induction of RNA toxicity (S3 Fig). The DM5^+/wt^/ *Prkca*^*-/-*^*/Prkcb*^*-/-*^ and a control group of DM5^+/wt^/ *Prkca*^*+/+*^*/Prkcb*^*+/+*^ mice did not show any evidence of myotonia by EMG) or cardiac conduction abnormalities (by ECG) prior to induction of RNA toxicity. The mice deficient for PKCα/PKCβ were slightly smaller, had a slightly longer PR-interval on ECG, and did not run as far on treadmill running assays, but showed no difference in grip strength (S1 Table).

**Fig 1:**
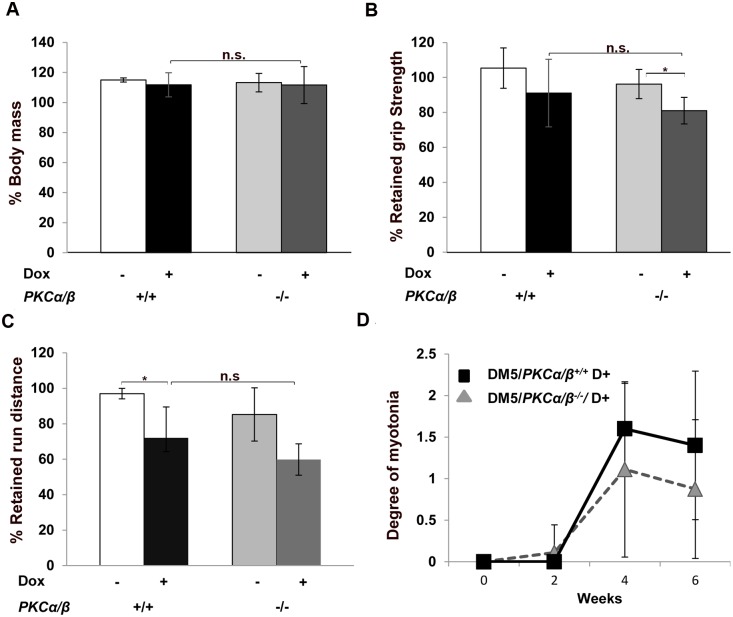
Muscle functions are not affected by *PKCα/β* deletion. (**A**) Body mass analysis showed no change in weight gain in uninduced and induced DM5/*PKCα/β* ^*-/-*^ and DM5/*PKCα/β* ^+/+^ mice. The graph shows body mass at 6 weeks post-induction expressed as the percentage of initial body mass. (**B, C**) At 6 weeks post-induction, no improvements in forelimb grip strength and treadmill running distances were found in DM5/*PKCα/β* ^*-/-*^ as compared to DM5/*PKCα/β* ^+/+^ in the RNA toxicity background. (**D**) Electromyography (EMG) analysis found no significant differences in myotonia between DM5/*PKCα/β* ^*-/-*^ and DM5/*PKCα/β* ^+/+^ after 2, 4, and 6 weeks post-induction of RNA toxicity. At least 4–5 mice were analyzed in each group. (*p = 0.05, Student’s t test); error bars are mean*±*stdev; n.s. means not significant.

After inducing the expression of the toxic RNA transgene (referred to as D+ or Dox+), mice were analyzed for body weight and tested by the aforementioned phenotypic assays at 2, 4, and 6 weeks post-induction. We found no change in body mass at 6-weeks post-induction between the two groups (Fig 1A). By six weeks post-induction, the DM5^+/wt^/ *Prkca*^*-/-*^*/Prkcb*^*-/-*^ mice became weaker but this was similar to the DM5^+/wt^/ *Prkca*^*+/+*^*/Prkcb*^*+/+*^ mice (Fig 1B). We tested these mice for their ability to run on a treadmill and recorded data as the percentage of retained run distance as compared to their pre-induction results. Again, we found that though both groups had deficits, there was no significant difference (Fig 1C). Also, no difference in cardiac conduction abnormalities were observed after 6 weeks of RNA toxicity (S3 Fig). Both groups of mice also developed a similar degree of myotonia by 4 or 6 weeks after induction of the toxic RNA (Fig 1D). We also confirmed that toxic RNA (S2 Fig) and *Clcn1* mRNA levels (S4 Fig) were similar between study groups by quantitative RT-PCR at 6 weeks post-induction. These results suggest that absence of PKCα/β has no beneficial effect on the muscle functions in these RNA toxicity mice.

### Absence of PKCα/β does not affect CELF1 expression in these RNA toxicity mice

Previous studies have shown that nuclear accumulation of the toxic RNA results in increased levels of CELF1 protein. The toxic RNA is thought to activate PKC signaling leading to CELF1 hyper-phosphorylation and stabilization [22]. Consistent with this idea, blocking PKC activity with Ro-31-8220 resulted in improvement in a heart-specific DM1 mouse model, and was correlated with reduced phosphorylation and decreased levels of CELF1 [23].

**Fig 2:**
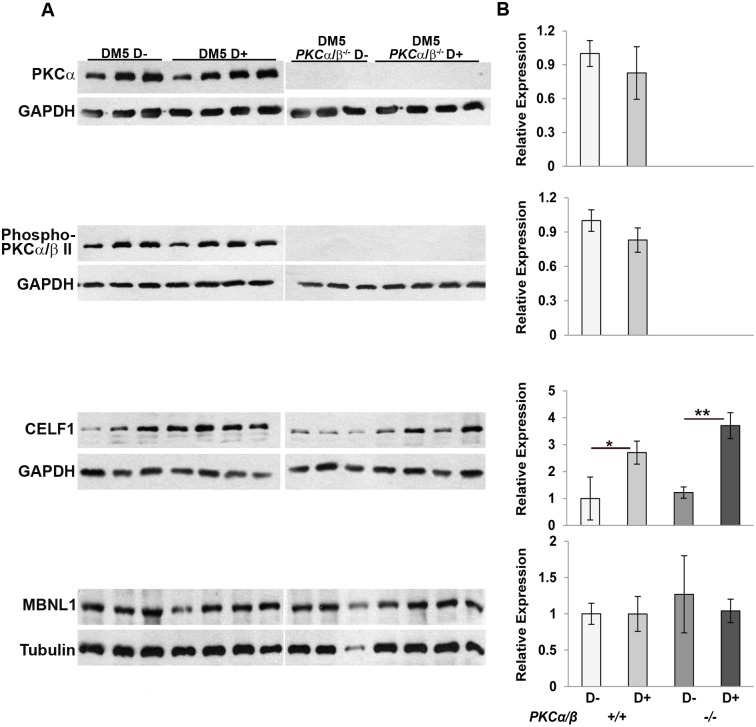
Expression of CELF1 is not affected by absence of PKCα/β. (**A**) Western blot analysis of relevant proteins from skeletal muscle extracts of DM5/*PKCα/β* ^*-/-*^ and DM5/*PKCα/β* ^+/+^ uninduced and induced mice at 6 weeks post-induction. Western blot analyses confirmed that PKCα and phospho-PKCα/β were absent in *PKCα/β* ^-/-^ mice. Western blot for CELF1 showed 2–4 fold induction in the RNA toxicity mice with or without PKCα/β. There was no difference in MBNL1 expression due to absence of PKCα/β or presence of RNA toxicity. GAPDH or Tubulin was used as a loading control. (**B**) Quantification of western blots is graphically depicted in right panel. At least 3–5 mice per group were used for analysis. A t-test was used to compare the results from uninduced and induced groups with or without PKCα/β. (*p = 0.05 Student’s t test); error bars are mean±stdev.

To see if the expression of CELF1 in skeletal muscle is also affected by PKCα/β in our RNA toxicity mice, we analyzed the expression of CELF1 in the *DM5*^*+/wt*^*/ Prkca*^*-/-*^*/Prkcb*^*-/-*^ (abbreviated as DM5 *PKCα/β*^*-/-*^) mice in the presence or absence of RNA toxicity. We first confirmed the absence of expression of PKCα/β and phospho-PKCα/β by western blot. Both PKCα and phospho-PKCα/β expression were absent in *DM5*^*+/wt*^*/ Prkca*^*-/-*^*/Prkcb*^*-/-*^ mice (Fig 2A). We then analyzed the expression of RNA binding proteins CELF1 and MBNL1 in induced DM5^+/wt^/ *Prkca*^*-/-*^*/Prkcb*^*-/-*^ mice as compared to induced DM5^+/wt^/ *Prkca*^*+/+*^*/Prkcb*^*+/+*^ mice. The expression of CELF1 is increased (2–3 fold) in DM5^+/wt^/ *Prkca*^*+/+*^*/Prkcb*^*+/+*^ D+ mice and its expression is still similarly high in the DM5^+/wt^/ *Prkca*^*-/-*^*/Prkcb*^*-/-*^ D+ mice, demonstrating that CELF1 is not modulated by PKCα/β in the skeletal muscles of these RNA toxicity mice. There were no differences in the expression of MBNL1 protein in any of these mice (Fig 2A and 2B).

### Mis-splicing and muscle histopathology in these RNA toxicity mice are not corrected by absence of PKCα

**Fig 3:**
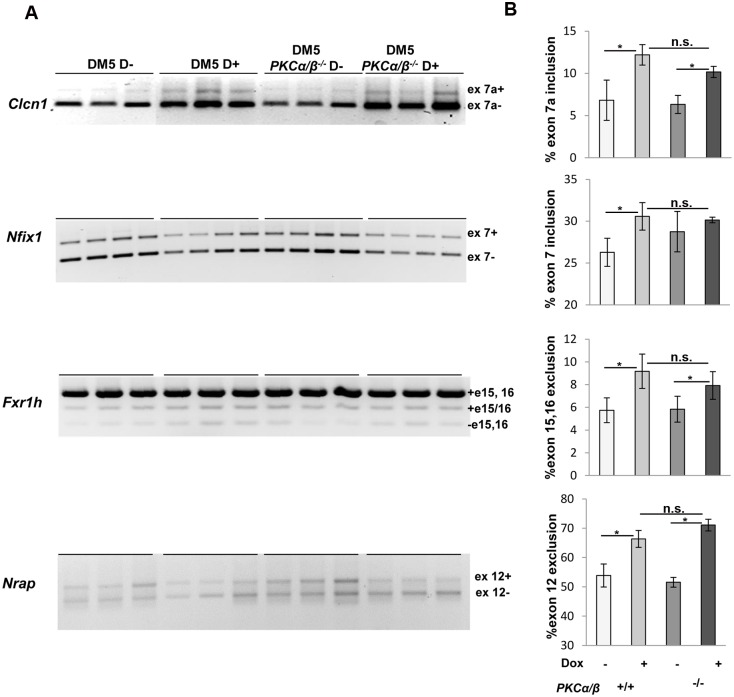
Absence of PKCα/β has no beneficial effects on RNA splicing defects caused by RNA toxicity. **(A)** RT-PCR analysis of several RNA splicing targets associated with RNA toxicity, *Clcn-1*(exon 7a), *Nfix1* (exon 7), *Fxr1h* (exon 15, 16), and *Nrap* (exon 12) shows that PKCα/β deficiency has no effect on splicing in the mice with RNA toxicity. (**B**) Quantification of the gels in (**A**) shows that RNA toxicity leads to splicing defects in the DM5 mice for all targets tested and PKCα/β deficiency has no effect on these splicing defects. For each groups, at least 4–5 mice were analyzed. *p = 0.05, Student’s t test; n.s. means not significant; error bars are mean±stdev.

To determine whether the absence of PKCα/β corrects the mis-splicing events affected by the toxic RNA, we analyzed splicing of *Clcn1* (ex7a), *Nfix1* (ex7), *Fxr1h* (exons 15, 16), and *Nrap* (exon 12), all targets which are mis-spliced in this mouse model of RNA toxicity [28]. All these targets were found to be misregulated by the toxic RNA in the *DM5*^*+/wt*^*/ Prkca*^*+/+*^*/Prkcb*^*+/+*^ mice (Fig 3A and 3B). But, we found similar levels of splicing defects in the *DM5*^*+/wt*^*/ Prkca*^*-/-*^*/Prkcb*^*-/-*^ mice in the presence of the toxic RNA. Although the splicing defects we studied were relatively mild, the absence of PKCα/β was still unable to correct these. The data suggests that these mis-splicing events in this RNA toxicity mouse model were independent of PKCα/β.

**Fig 4:**
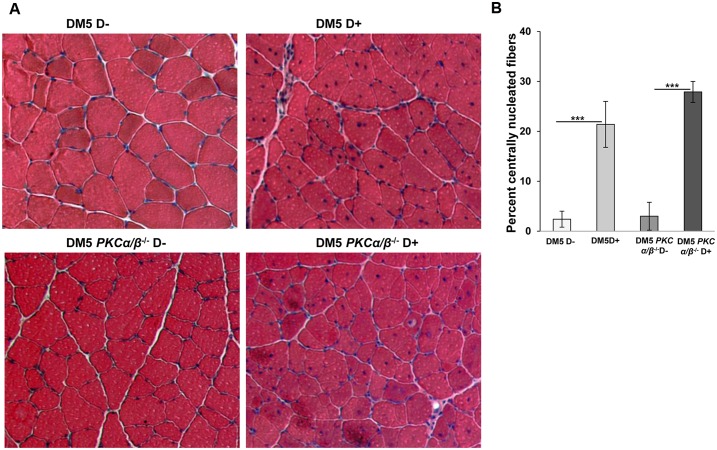
Muscle histopathology in the RNA toxicity mice is not corrected by absence of PKCα/β. (**A**) Hematoxylin and eosin (H&E) staining was performed on quadriceps muscles form DM5/*PKCα/β* ^*-/-*^ and DM5/*PKCα/β* ^+/+^ mice in the absence or presence of RNA toxicity. A representative H&E section is shown for each group. Uninduced DM5/*PKCα/β* ^*-/-*^ and DM5/*PKCα/β* ^+/+^ mice showed normal muscle histology. By 6 weeks of 0.02% doxycycline induction, we saw similar level of histopathology in the presence or absence of PKCα/β. (**B**) In the RNA toxicity mice; ~20% of nuclei were centralized. No significant difference was observed in the percentage of centrally nuclei between DM5/*PKCα/β* ^*-/-*^ D+ and DM5/*PKCα/β* ^+/+^ D+ mice. For each groups, at least 4–5 mice were analyzed. At least 300 fibers were analyzed per mouse. ***p = 0.001, Student’s t test; error bars are mean±stdev.

**Fig 5:**
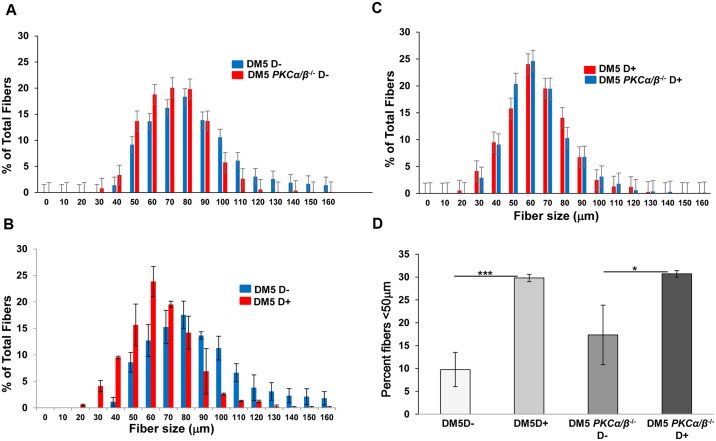
*PKCα/β* deficiency does not affect skeletal muscle fiber size distribution in the RNA toxicity mice. (**A**) Histogram of skeletal muscle fiber diameter sizing shows that *PKCα/β* deficiency in the absence of RNA toxicity mice (DM5/*PKCαβ*^*-/-*^ D- (n = 3)) (red) results in a distribution similar to that seen in uninduced mice (DM5/*PKCαβ*^*+/+*^ D- (n = 3)) (blue). (**B**) Histogram of skeletal muscle fiber diameter distribution shows that smaller fibers in the RNA toxicity mice (DM5 D+ (n = 3)) (red) as compared to uninduced mice (DM5 D- (n = 3)) (blue). (**C**) Histogram of skeletal muscle fiber diameter sizing shows that no differences in fiber size distribution after *PKCα/β* deficiency (blue) in the presence of RNA toxicity (red). (**D**) The graph showing no differences in the percentage of fibers less than 50μm in the *PKCα/β* deficiency mice as compared to normal mice in the presence of RNA toxicity. Note the increased percentage fibers in the presence of toxic RNA as compared to uninduced mice. At least 300 fibers were analyzed per mouse. *p = 0.05, and ***p = 0.001, Student’s t test; error bars are mean±stdev.

To investigate the muscle histopathology, quadriceps muscles from uninduced and induced DM5^+/wt^/ *Prkca*^*-/-*^*/Prkcb*^*-/-*^ and *DM5*^*+/wt*^*/ Prkca*^*+/+*^*/Prkcb*^*+/+*^ mice were stained with hematoxylin and eosin. These mice showed normal histology with uniform fiber size in the absence of the toxic RNA. After 0.02% doxycycline induction, DM5^+/wt^/ *Prkca*^*-/-*^*/Prkcb*^*-/-*^ mice had various histological features including increased central nuclei and variation in fiber size similar to *DM5*^*+/wt*^*/ Prkca*^*+/+*^*/Prkcb*^*+/+*^ mice (Fig 4A). No significant differences in the number of central nuclei per fiber were found between the two groups of mice (Fig 4B). Analyses of the fiber size distribution in skeletal muscles from the DM5^+/wt^/ *Prkca*^*-/-*^*/Prkcb*^*-/-*^ D-yielded similar distribution to those of *DM5*^*+/wt*^*/ Prkca*^*+/+*^*/Prkcb*^*+/+*^ D- (Fig 5A). With RNA toxicity, the fiber size distribution was significantly altered in *DM5*^*+/wt*^*/ Prkca*^*+/+*^*/Prkcb*^*+/+*^ D+ mice as compared to *DM5*^*+/wt*^*/ Prkca*^*+/+*^*/Prkcb*^*+/+*^ D- mice (Fig 5B). But, there were no significant differences in muscle fiber distribution upon deletion of PKCα/β in the presence of the toxic RNA (Fig 5C). Similarly, no significant differences in muscle fiber diameter (<50 μm) were observed (Fig 5D). The data suggest that PKCα/β deficiency is not beneficial to histopathology caused by the RNA toxicity.

## Discussion

Previously, we reported that expression of the toxic RNA in our mouse model results in many features of DM1 includes myotonia, abnormal muscle pathology, and RNA splicing defects [27]. Using this mouse model, we have shown that CELF1 is post-transcriptionally increased in response to the toxic RNA and that CELF1 contributes to skeletal muscle histopathology [28]. In that study, we found that depletion of CELF1 stabilized some functional phenotypes and improved skeletal histopathology in the RNA toxicity mice [28].

However, the roles of PKCα/β, which have been reported to increase CELF1 levels through phosphorylation and increased protein stability [22], have not been investigated in the skeletal muscle of mice with RNA toxicity. In this study, we used a clear genetic approach to eliminate the expression of both PKCα and PKCβ in these mice with RNA toxicity. We find that key muscle phenotypes associated with RNA toxicity are independent of PKCα/β. We also show that increased CELF1 levels are not mitigated by the absence of PKCα/β in skeletal muscle. Concordantly, neither functional outcomes nor abnormal muscle histology in our mice expressing the toxic RNA were restored towards normal in the absence of PKCα/β.

Both PKCα and PKCβ have been implicated in the pathogenesis of cardiac disease with pharmacological and gene-therapy based inhibition of PKCα/β having been shown to enhance cardiac contractility in heart failure models [29, 32]. With respect to RNA toxicity associated with DM1, previous studies have reported that PKCα/β signaling is activated in cells expressing expanded CUG repeat RNAs and that PKCα/β inhibition by Ro-31-8220 correlates with reduced PKCα/β activation and CELF1 levels [22]. In a cardiac specific mouse model, treatment with Ro-31-8220 was associated with improved cardiac function and also attenuated splicing defects related to increased CELF1 levels [22, 23]. In contrast, using a genetic approach, we find that PKCα/β is not involved in affecting skeletal muscle phenotypes in our RNA toxicity mice.

Failure to rescue the phenotypes in our RNA toxicity models by genetic deletion of PKCα/β could be attributed to differences in the mouse model and the approaches used in the different studies. Using a human *DMPK* promoter, our mouse model expresses its toxic RNA in multiple tissues that are affected in DM1 including skeletal muscle, the heart and smooth muscle [27]. The other published models have used non-*DMPK* tissue specific promoters [22, 23]. Our mouse model has DM1 relevant phenotypes such as myotonia, cardiac conduction defects, RNA splicing defects, increased CELF1 in skeletal muscle, muscle histopathology and shortened lifespan (likely due to cardiac conduction abnormalities) that are present simultaneously and clearly responsive to RNA toxicity. Limited subsets of these phenotypes are also seen in a tissue specific manner in the other mouse models. In addition, as in the other mouse models, MBNL1 does bind the RNA expressed in our mice [33]. However, our mice express a *DMPK* 3'UTR RNA with (CUG)_5_ (i.e. a perfect but non-expanded repeat tract) that does not form visible RNA foci, and the other mouse models express a RNA comprising of a concatemer of forty eight, interrupted but non-expanded repeat tracts containing (CUG)_20_ that forms visible RNA foci. Whether RNA foci play a role in affecting CELF1 levels is uncertain since it has been reported that the HSA-LR mice (which express only an expanded (CUG)_250_) with many RNA foci and DM2 patients whose RNA foci contain only expanded (CCUG)_n_, do not show increased CELF1 in skeletal muscle [34] It may be that these distinctions account for the differences in the various studies.

It is interesting to note that Ro-31-8220 did not influence the phenotype of a mouse model engineered to over-express CELF1, despite the fact that this model recapitulated aspects of DM1 [23]. Thus, over-expression of CELF1 may cause DM1 associated phenotypes in a PKC independent manner. This is analogous to our observations. It is also possible that DM1 phenotypes induced by RNA toxicity are inhibited by compounds such as Ro-31-8220 through a variety of means. Although Ro-31-8220 has stated potency against PKCα, it can also affect other kinases including GSK3β [35–37]. The results of our study with the deletions of PKCα and PKCβ clearly demonstrate that alternate pathways are likely involved in CELF1 regulation in our mice. Similarly, a recent study demonstrated that the effect of Ro-31-8220 on the toxic RNA in DM1 cells may be independent of effects on PKCα [38]. CELF1 has been posited as a substrate for a number of kinases including Akt, cyclinD3/cdk [39] and more recently, GSK3β [21]. In investigating some of these other targets, we find that GSK3β levels are increased in the skeletal muscles of our mice with RNA toxicity (S5 Fig). Though they are not part of this study, results such as these provide fertile ground for future studies assessing the effects of pathways such as GSK3β and the role that inhibitors such as the Ro-81-3220 may play.

**Fig 6:**
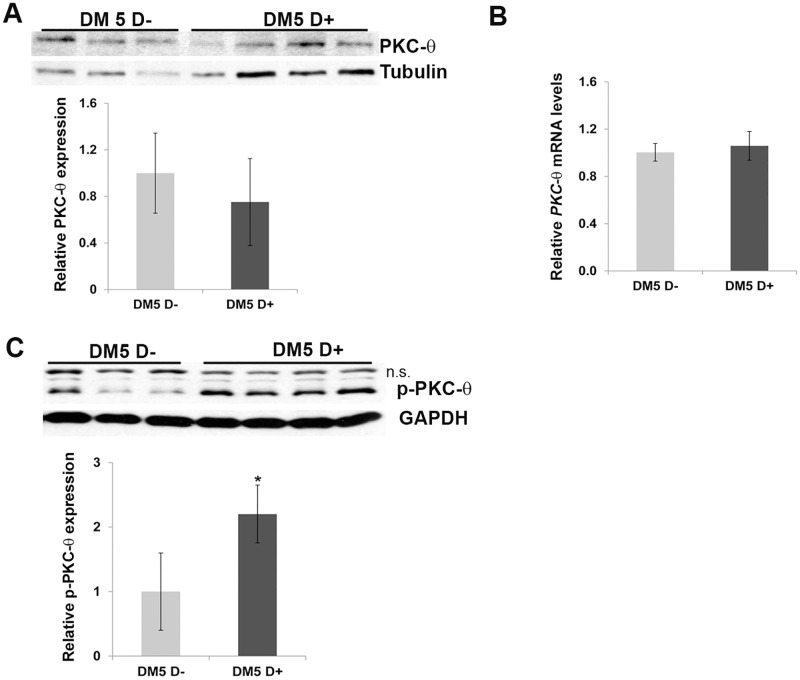
Phospho-PKCθ levels are increased in the RNA toxicity mice. (**A, B**) Western blot and quantitative RT-PCR shows no significant differences in PKCθ expression in uninduced and induced skeletal muscle tissues from RNA toxicity mice (n = 3–5 for uninduced and n = 3–5 for induced mice). Tubulin-loading control. (**C**) Western blot of skeletal muscle protein extracts shows increased levels of p-PKCθ in DM5 mice with RNA toxicity. n.s. indicates non-specific band; GAPDH-loading control. *p = 0.05, Student’s t test; error bars are mean±stdev.

The protein kinase C family comprises at least 10 different isozymes, which are classified by their second messenger activators [24]. The predominant forms reported to be expressed in skeletal muscle are PKCα and PKCθ [25]. PKCα accounts for approximately 97% of the classical PKC activity and, has primarily been studied in skeletal muscle with respect to its effects on glucose metabolism and insulin responsiveness [26, 30]. Interestingly, PKCθ (PKC-theta), another isoform expressed in skeletal muscle has been suggested to be involved in a number of biological events and phenotypes that have been associated with DM1. For instance, PKCθ has been suggested to plays role in myoblast fusion [40, 41], a role in modulating chloride channel function in skeletal muscle [42] and its loss has been shown to reduce skeletal muscle histopathology in a Duchenne Muscular Dsytrophy (DMD) mouse model [43, 44]. Given our negative results with PKCα/β, we investigated the levels of PKCθ and found that phosphorylated PKCθ levels were increased in the skeletal muscles of our RNA toxicity mice (Fig 6, S6 Fig).

In conclusion, using double knockout mice for PKCα and PKCβ, we find that PKCα/β are not required for the skeletal muscle phenotypes in our RNA toxicity mice. Our data also suggest that PKCα/β play little role in increased CELF1 levels and or the aberrant splicing events observed in the skeletal muscles of our mouse model. Interestingly our preliminary evaluation of alternative kinases that might be involved in RNA toxicity suggests that a previously reported target, GSK3β, and a novel target, PKCθ, are both affected in our mouse model and warrant further investigation in future studies.

## Materials and Methods

### Animal models

All animals were used in accordance with protocols approved by the Animal Care and Use Committee at the University of Virginia. The RNA toxicity mice (DM5) were described previously [27, 45]. Of note, they express an RNA with a (CUG)_5_ tract. One *Pkcα* knockout mouse (*Prkca*^*tm1Jmk*^) was obtained from Dr. J. Molkentin [29]. We also obtained another *Pkcα* knockout mouse and *Pkcβ* knockout mice from Dr. M. Leitges [30, 31]. The majority of the experiments were done with the latter mice. DM5 mice were mated with *PKCα*^*-/-*^ (*Prkca*^*-/-*^) and *PKCβ*^*-/-*^ (*Prkcb*^*-/-*^) mice to generate DM5/*PKCα/β* ^*-/-*^ and DM5/*PKCα/β* ^+/+^ mice. The transgenic mice were induced with 0.02% doxycycline in drinking water.

### Western blot analysis

Protein extracts were made using standard protocols in RIPA buffer (50 mM Tris-HCl, pH 7.4, 150 Mm NaCl, 1%NP40, 0.5% Na-deoxycholate, and 0.1% sodium dodecyl sulfate (SDS) and protease inhibitor (Roche Inc., cat. #1873580). Proteins were detected with the following antibodies: CELF1 (3B1, EMD Milipore), PKCα (Santa Cruz Biotechnology, SC-208), p-PKCα/β (Cell signaling, #9375), GSK-3β (Santa Cruz Biotechnology, SC-71186), Phospho-PKCθ (Cell signaling, #9377), PKCθ (Cell Signaling, #12206), GAPDH (Ambion #4300), MBNL1 (A2764, gift from Dr. Charls A. Thornton), Tubulin (Sigma-Aldrich #T6199).

### Phenotypic analysis

Mice were analyzed for running on treadmill and forelimb grip strength was measured using a digital grip-strength meter. All the details about protocols are described elsewhere [28]. All results are reported as retained function with reference to baseline for each mouse. EMG and ECG were also measured as described previously [28].

### RNA isolation, qRT-PCR assays and splicing analysis

Total RNA was extracted from skeletal muscle tissues using protocol as described [46]. 1 μg of total RNA was used for making cDNA using QuantiTech Reverse Transcription Kit (Qiagen). qRT-PCR was done using the BioRad iCycler and detected with SYBER-Green dye. Data were normalized using endogenous control (*Gapdh*), and normalized values were subjected to a 2^-ΔΔCt^ formula to calculate the fold changes between uninduced and induced groups. Primer sequences are given in S2 Table. All the splicing assays were done in at least five mice or more per group. Splicing primers and conditions have been described in S3 Table.

### Histology and fiber size quantitation

H&E staining was done according to standard procedures and examined under a light microscope. Histopathology was assessed by H&E staining of quadriceps femoris (6 μm) cryosections. Muscle fiber size was determined using AxioVision^™^ V4.8.2.0 (Carl Zeiss MicroImaging). At least 3–5 mice per group were studied and for each mouse, 3–5 images were analyzed.

### Statistical analysis

Statistical significance was determined using a two-tailed Student’s t-test with equal or unequal variance as appropriate. All data are expressed as mean ± standard deviation. p<0.05 was considered statistically significant unless otherwise specified.

### Study Approvals

All animal protocols were approved by the institutional ICAUC at the University of Virginia.

## Supporting Information

S1 Fig Phenotypic Analyses in DM5 *PKC*^*-/-*^ (DM5/*Prkca*^*tm1Jmk-/-*^) mice. **(A)** survival percentages, **(B)** % retained run distance and **(C)** % retained grip strength. DM5 *PKCα*^*+/+*^ (DM5/*Prkca*^*tm1Jmk+/+*^) (n = 24), DM5 *PKCα*^*+/-*^ (DM5/*Prkca*^*tm1Jmk+/-*^) (n = 27), and DM5 *PKC*^*-/-*^ (DM5/*Prkca*^*tm1Jmk-/-*^) (n = 15).
(TIF)

S2 Fig Levels of toxic RNA are not affected by *PKCα/β* deficiency. Quantitative RT-PCR of eGFP mRNA show no difference in the levels of toxic RNA and *Clcn1* between DM5 mice that are wildtype for *PKCα/β* and those that have *PKCα/β* deleted. *p = 0.05, and **p = 0.01, Student’s t test; error bars are mean±SEM.
(TIF)

S3 Fig P-R intervals are not affected by *PKCα/β* deficiency. Cardiac conduction abnormalities (by ECG) show no significant differences in P-R interval with or without PKCα/β after induction of RNA toxicity for up to 8 weeks. At least n = 5/group used for analysis.
(TIF)

S4 Fig Levels of *Clcn1* mRNA are not affected by *PKCα/β* deficiency. Quantitative *Clcn1* mRNA shows no difference in the levels of *Clcn1* between DM5 mice that are wildtype for *PKCα/β* and those that have *PKCα/β* deleted. *p = 0.05, and **p = 0.01, Student’s t test; error bars are mean±SEM.
(TIF)

S5 Fig Increased GSK-3β in the RNA toxicity mice. **(A)** Western blot of skeletal muscle protein extracts shows increased GSK-3β in skeletal muscle of DM5- (D+) mice. Relative levels indicated below. **(B)** Quantitative RT-PCR shows increased GSK-3β the mice with RNA toxicity. (At least n = 5/group used for analysis). **p = 0.008, Student’s t test; error bars are mean±stdev.
(TIF)

S6 Fig Phospho-PKCθ levels are increased in the DM200 mice. Western blot of skeletal muscle protein extracts shows increased levels of p-PKCθ in DM200 mice with RNA toxicity. GAPDH as loading control.
(TIF)

S1 Table Phenotypic analysis of DM5^+/wt^/ *Prkca*^*-/-*^*/Prkcb*^*-/-*^ and DM5^+/wt^/ *Prkca*^*+/+*^*/Prkcb*^*+/+*^ uninduced mice. (DOCX)

S2 Table Primers for RT-PCR assays. (DOCX)

S3 Table Primers for splicing assays. (DOCX)
