# Supplementary figures and images for: Disease Phenotypes in a Mouse Model of RNA Toxicity Are Independent of Protein Kinase Cα and Protein Kinase Cβ
Source: PLoS One. 2016 Sep 22;11(9):e0163325. doi: 10.1371/journal.pone.0163325 (PMC5033491; doi:10.1371/journal.pone.0163325)

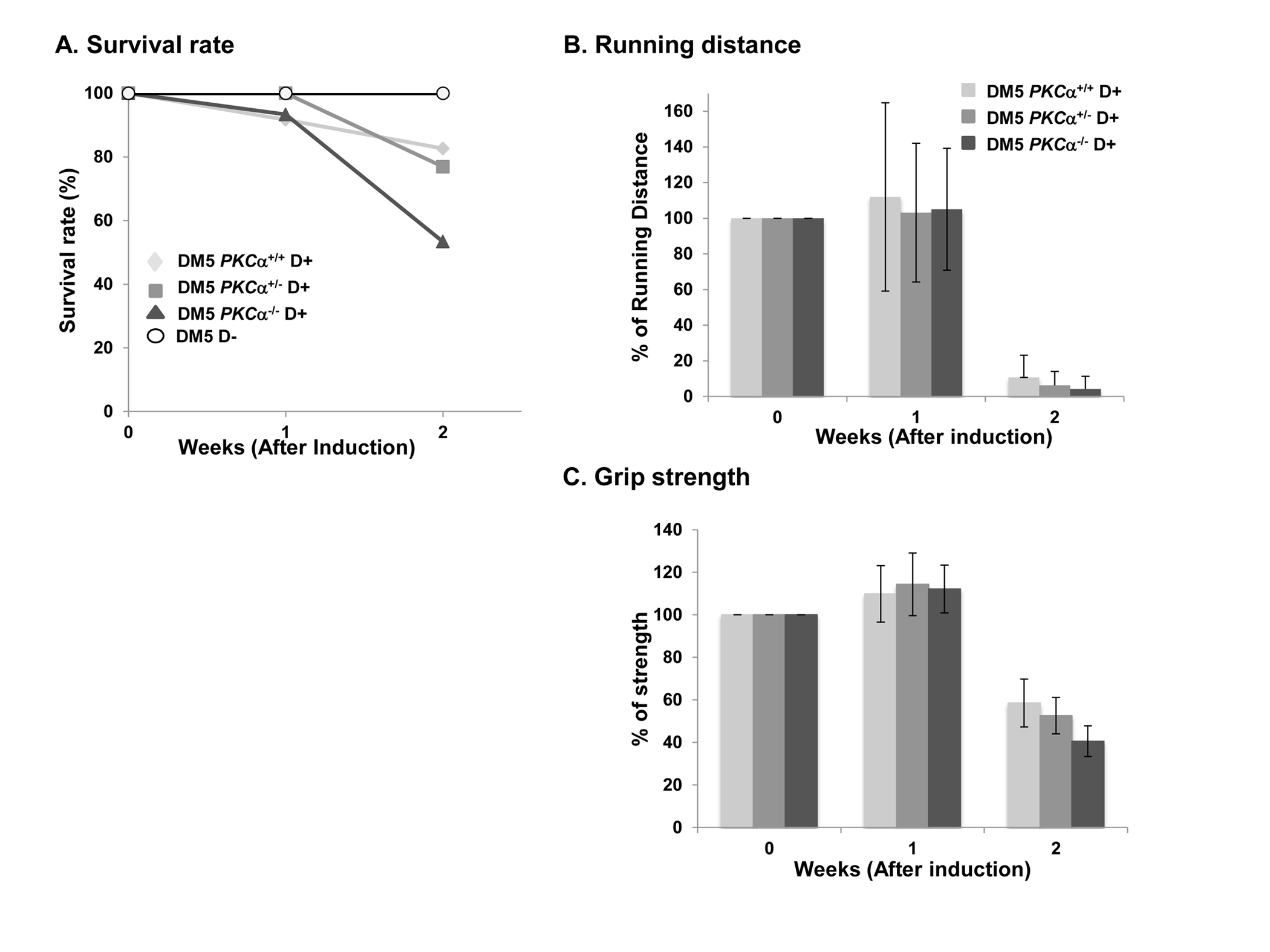

Supplement: S1 Fig — (A) survival percentages, (B) % retained run distance and (C) % retained grip strength. DM5 PKCα+/+ (DM5/Prkcatm1Jmk+/+) (n = 24), DM5 PKCα+/- (DM5/Prkcatm1Jmk+/-) (n = 27), and DM5 PKC-/- (DM5/Prkcatm1Jmk-/-) (n = 15). (TIF) [file pone.0163325.s001.tif]

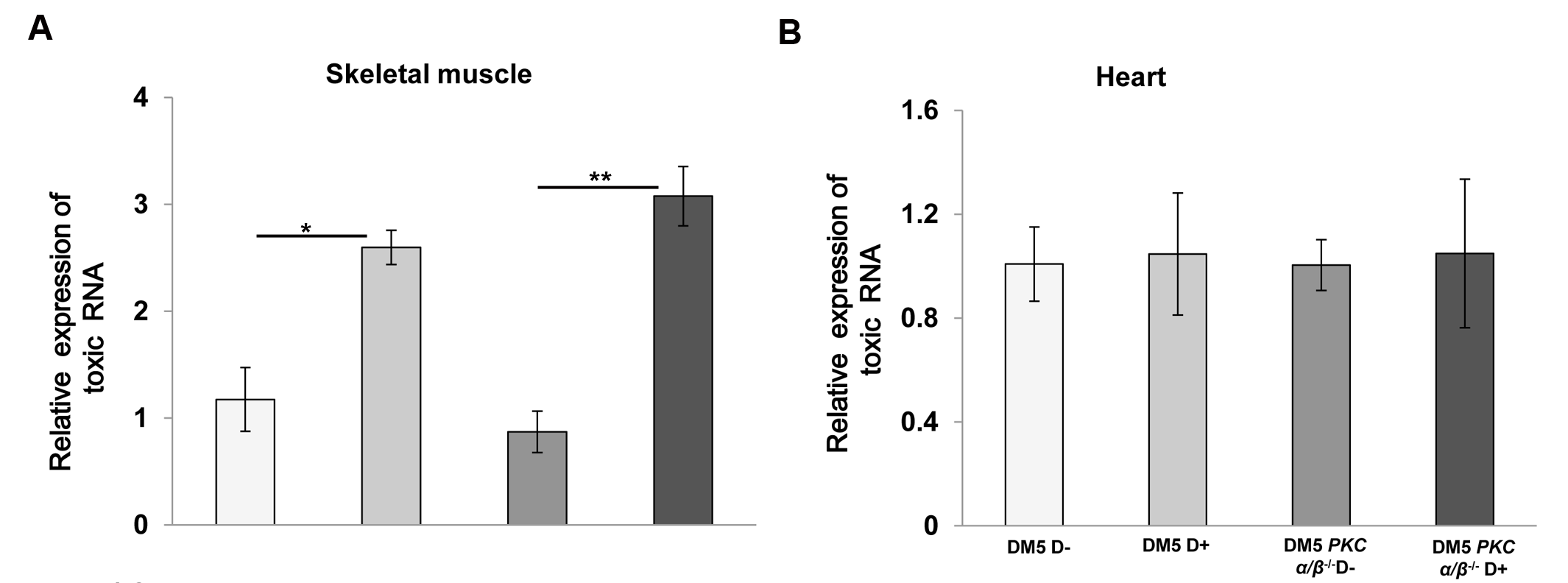

Supplement: S2 Fig — Quantitative RT-PCR of eGFP mRNA show no difference in the levels of toxic RNA and Clcn1 between DM5 mice that are wildtype for PKCα/β and those that have PKCα/β deleted. *p = 0.05, and **p = 0.01, Student’s t test; error bars are mean±SEM. (TIF) [file pone.0163325.s002.tif]

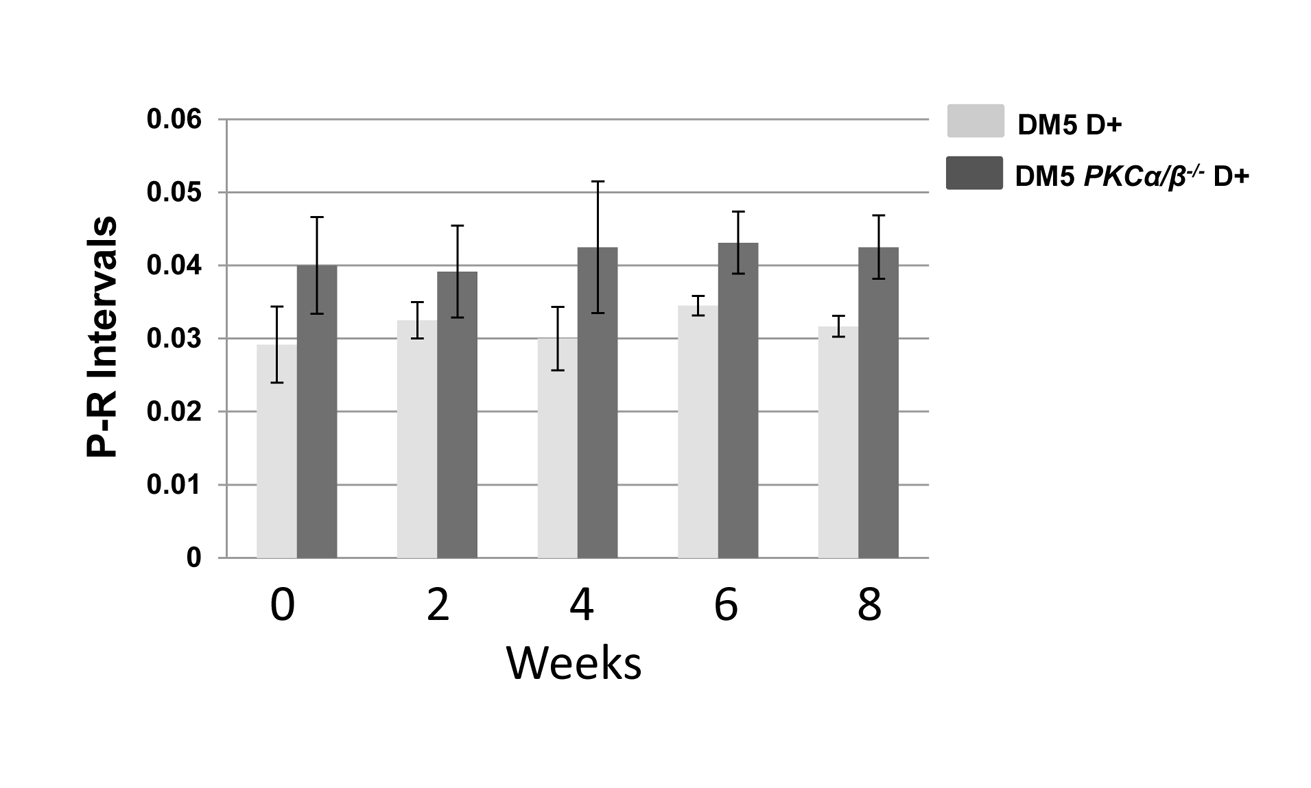

Supplement: S3 Fig — Cardiac conduction abnormalities (by ECG) show no significant differences in P-R interval with or without PKCα/β after induction of RNA toxicity for up to 8 weeks. At least n = 5/group used for analysis. (TIF) [file pone.0163325.s003.tif]

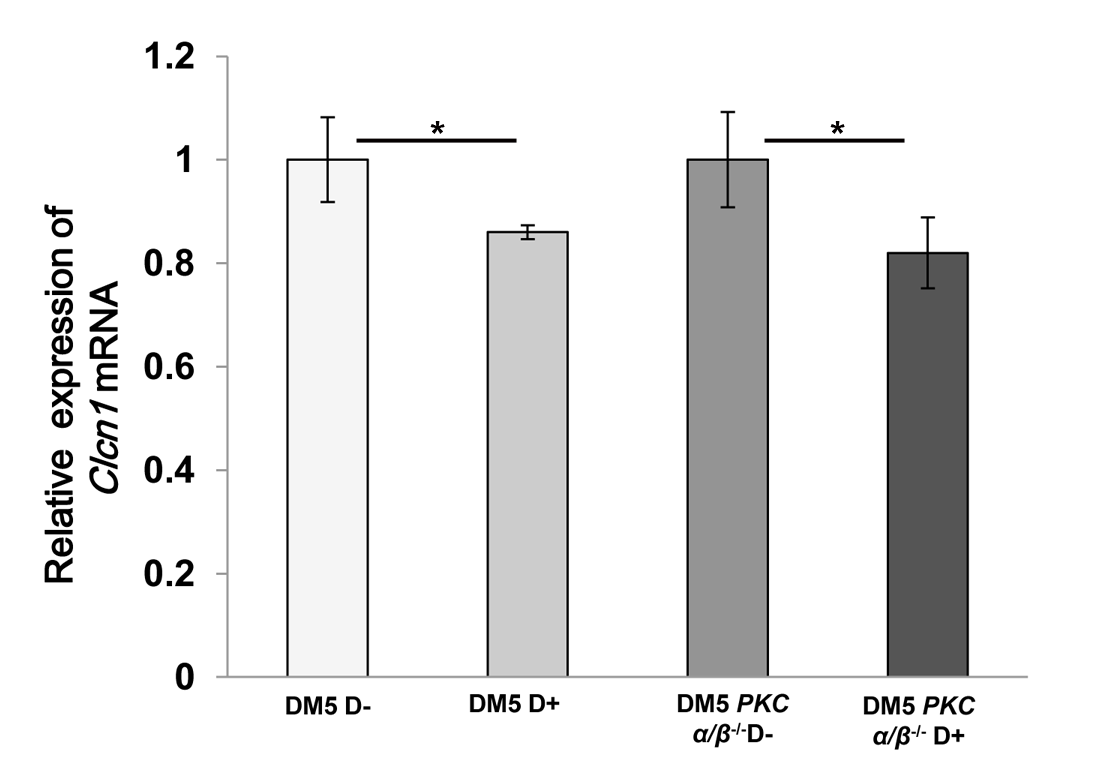

Supplement: S4 Fig — Quantitative Clcn1 mRNA shows no difference in the levels of Clcn1 between DM5 mice that are wildtype for PKCα/β and those that have PKCα/β deleted. *p = 0.05, and **p = 0.01, Student’s t test; error bars are mean±SEM. (TIF) [file pone.0163325.s004.tif]

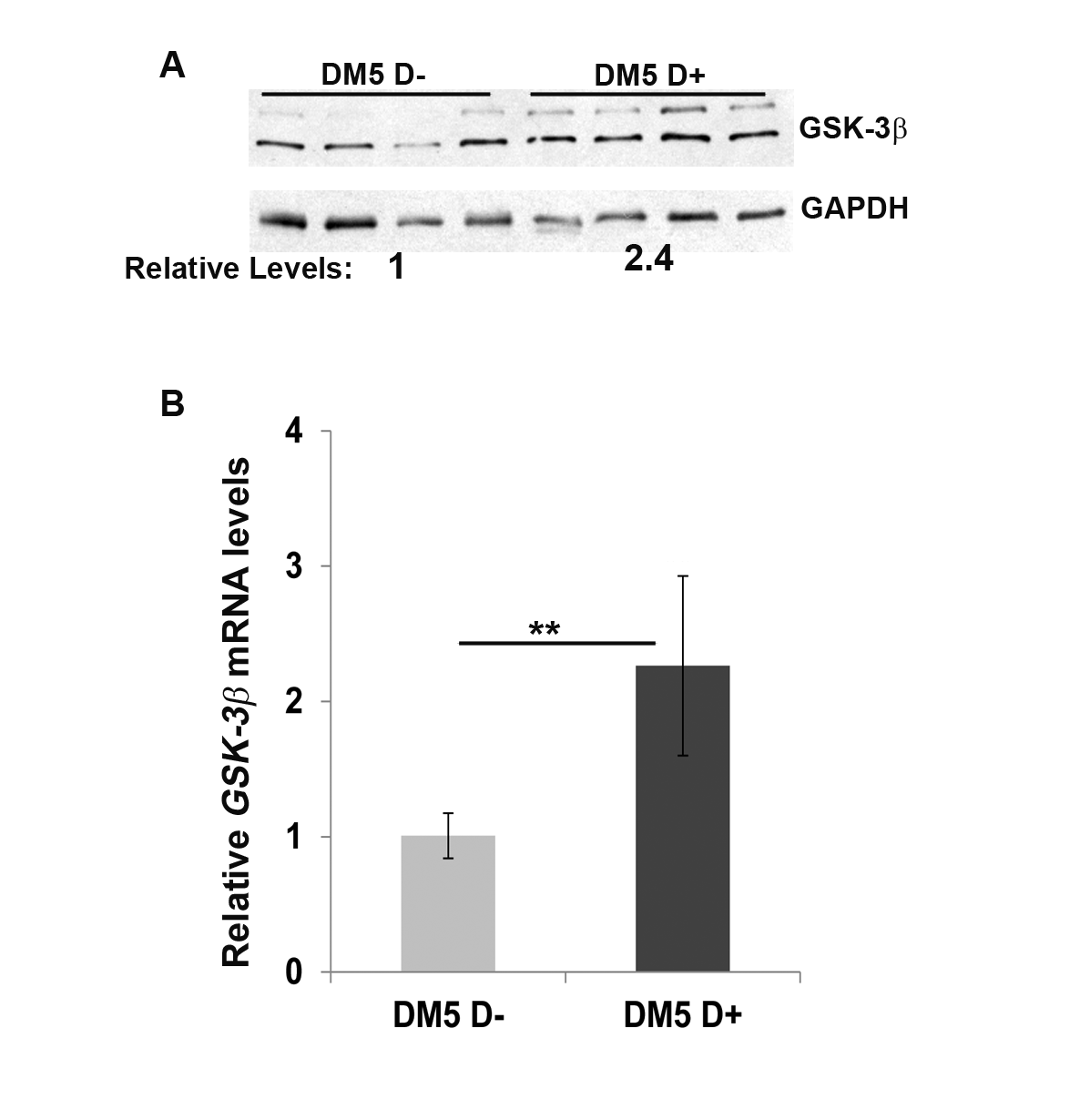

Supplement: S5 Fig — (A) Western blot of skeletal muscle protein extracts shows increased GSK-3β in skeletal muscle of DM5- (D+) mice. Relative levels indicated below. (B) Quantitative RT-PCR shows increased GSK-3β the mice with RNA toxicity. (At least n = 5/group used for analysis). **p = 0.008, Student’s t test; error bars are mean±stdev. (TIF) [file pone.0163325.s005.tif]

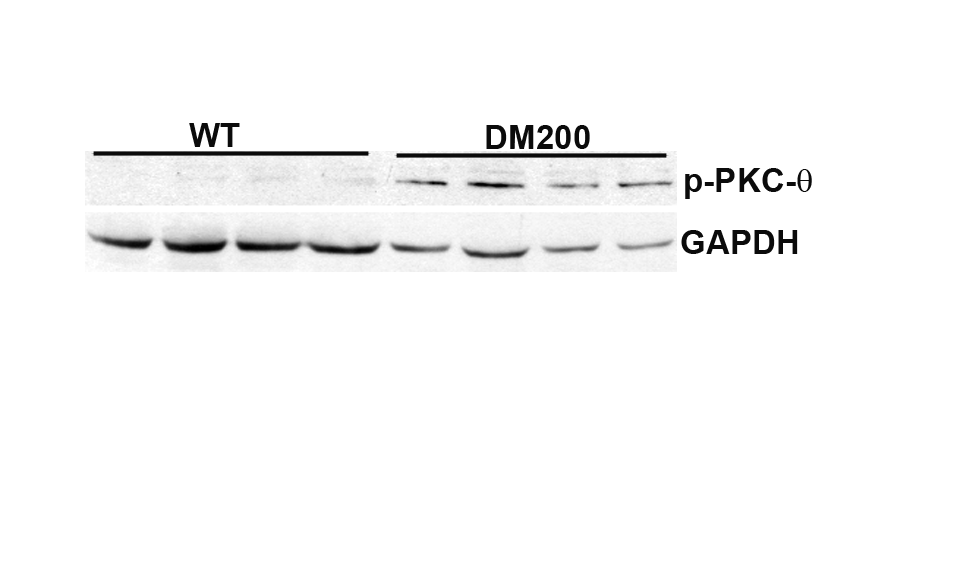

Supplement: S6 Fig — Western blot of skeletal muscle protein extracts shows increased levels of p-PKCθ in DM200 mice with RNA toxicity. GAPDH as loading control. (TIF) [file pone.0163325.s006.tif]
