# Supplementary material for: Disease Phenotypes in a Mouse Model of RNA Toxicity Are Independent of Protein Kinase Cα and Protein Kinase Cβ
Source: PLoS One. 2016 Sep 22;11(9):e0163325. doi: 10.1371/journal.pone.0163325 (PMC5033491; doi:10.1371/journal.pone.0163325)
Supplement: S1 Table — (DOCX) [file pone.0163325.s007.docx]

**S1 Table.** **Phenotypic analysis of DM5^+/wt^/ *Prkca^-/-^/Prkcb^-/-^*** **and DM5^+/wt^/ *Prkca^+/+^/Prkcb^+/+^* uninduced mice.**

| **Phenotypes** | **DM5/ *Prkca^-/-^/Prkcb^-/-^*(n=15)** | | **DM5/*Prkca^+/+^/Prkcb^+/+^* (n=10)** | | **ttest** |
| --- | --- | --- | --- | --- | --- |
| Weight | 20.75±2.54 | | 23.01±2.57 | | 0.04 |
| Run distance | 201±65 | | 752±0 | | <0.0001 |
| Grip strength | 87±12 | | 91±15 | | 0.35 |
| EMG | 0 | | 0 | | - |
| ECG | | 0.038±0.0046 | | 0.033±0.0057 | 0.02 |
