# Supplementary material for: Disease Phenotypes in a Mouse Model of RNA Toxicity Are Independent of Protein Kinase Cα and Protein Kinase Cβ
Source: PLoS One. 2016 Sep 22;11(9):e0163325. doi: 10.1371/journal.pone.0163325 (PMC5033491; doi:10.1371/journal.pone.0163325)
Supplement: S2 Table — (DOCX) [file pone.0163325.s008.docx]

**S2 Table.** **Primers for real-time PCR.**

| **Gene** | **Forward primer** | **Reverse primer** | **Anneal-**  **ing temp (°C)** | **PCR effi-cien-cy (%)** |
| --- | --- | --- | --- | --- |
| *GFP* | 5’GGGCACAAGCTGGAGTACAACTAC | 5’-ACTTGTACAGCTCGTCCATGC | 62 | 97 |
| *Clcn1* | 5’-CTCCCTTCCAGCTGGTGGAG | 5’-CTAGTGCCAAGACACCTCTGAGC | 55 | 97 |
| *Gapdh* | 5’-AGGTCGGTGTGAACGGATTTG | 5’TGTAGACCATGTAGTTGAGGTCA | 62 | 93 |
| *PKC-Ө* | 5’-GTTAGAGCTGAAACCTCAAGG | 5’-TGGCAGACAGAGCAAAATGTG | 61 | 97 |
| *GSK-3β* | 5’-TGGCAGCAAGGTAACCACAG | 5’CGGTTCTTAAATCGCTTGTCCTG | 61 | 95 |
