# Supplementary material for: Disease Phenotypes in a Mouse Model of RNA Toxicity Are Independent of Protein Kinase Cα and Protein Kinase Cβ
Source: PLoS One. 2016 Sep 22;11(9):e0163325. doi: 10.1371/journal.pone.0163325 (PMC5033491; doi:10.1371/journal.pone.0163325)
Supplement: S3 Table — (DOCX) [file pone.0163325.s009.docx]

**S3 Table.** **Primers for splicing assays.**

| ***Splicing primers*** | **Forward primer** | **Reverse primer** | **Anneal-**  **ing temp (°C)** | **Cycle** |
| --- | --- | --- | --- | --- |
| *Clcn1* | 5’-GCTGCTGTCCTCAGCAAGTT | 5’-CTGAATGTGGCTGCAAAGAA | 58 | 30x |
| *Nfix1* | 5’-TCGACGACAGTGAGATGGAG | 5’-CAAACTCCTTCAGCGAGTCC | 55 | 30x |
| *Fxr1h* | 5’-GATAATACAGAATCCGATCAG | 5’CTGAAGGACCATGCTCTTCAATCAC | 57 | 28x |
| *Nrap* | 5’GACCGATGTGGCCAGGTTTACTCA | 5’CAGGGGAACCAGCCTCATCGTTGTTTG | 64 | 30x |
